# Identification of GALNT14 as a novel neuroblastoma predisposition gene

**DOI:** 10.18632/oncotarget.4501

**Published:** 2015-07-03

**Authors:** Marilena De Mariano, Roberta Gallesio, Marco Chierici, Cesare Furlanello, Massimo Conte, Alberto Garaventa, Michela Croce, Silvano Ferrini, Gian Paolo Tonini, Luca Longo

**Affiliations:** ^1^ U.O.C. Bioterapie, IRCCS A.O.U. San Martino-IST, Istituto Nazionale per la Ricerca sul Cancro, Genoa, Italy; ^2^ Fondazione Bruno Kessler, Trento, Italy; ^3^ G. Gaslini Institute, Genoa, Italy; ^4^ Neuroblastoma Laboratory, Pediatric Research Institute, Fondazione Città della Speranza, Padua, Italy

**Keywords:** neuroblastoma, genetic predisposition, GALNT14, exome-sequencing, O-glycosylation

## Abstract

Although several genes have been associated to neuroblastoma (NB) predisposition and aggressiveness, further genes are likely involved in the overall risk of developing this pediatric cancer. We thus carried out whole-exome sequencing on germline DNA from two affected second cousins and two unlinked healthy relatives from a large family with hereditary NB. Bioinformatics analysis revealed 6999 variations that were exclusively shared by the two familial NB cases. We then considered for further analysis all unknown or rare missense mutations, which involved 30 genes. Validation and analysis of these variants led to identify a *GALNT14* mutation (*c.802C > T*) that properly segregated in the family and was predicted as functionally damaging by PolyPhen2 and SIFT. Screening of 8 additional NB families and 167 sporadic cases revealed this *GALNT14* mutation in the tumors of two twins and in the germline of one sporadic NB patient. Moreover, a significant association between *MYCN* amplification and *GALNT14* expression was observed in both NB patients and cell lines. Also, *GALNT14* higher expression is associated with a worse OS in a public dataset of 88 NB samples (http://r2.amc.nl). *GALNT14* is a member of the polypeptide N-acetylgalactosaminyl-transferase family and maps closely to *ALK* on 2p23.1, a region we previously discovered in linkage with NB in the family here considered. The aberrant function of GALNTs can result in altered glycoproteins that have been associated to the promotion of tumor aggressiveness in various cancers. Although rare, the recurrence of this mutation suggests *GALNT14* as a novel gene potentially involved in NB predisposition.

## INTRODUCTION

Neuroblastoma (NB) is a cancer of the sympathetic nervous system, which accounts for about 10% of all pediatric tumors and 15% of childhood cancer mortality. The prevalence is about one case in 7,000 live births [1], and approximately 120 new cases are diagnosed each year in Italy. Although it is relatively rare, NB is the third pediatric cancer after leukemias and tumors of the central nervous system, and the first solid tumor in children in the pre-scholar age [1, 2]. NB arises from neural crest derivatives that are committed to origin chromaffin cells in the medulla of the adrenal gland and paraspinal sympathetic ganglia in the neck, chest or abdomen, or in pelvic ganglia [1]. Despite intensive multimodality treatments NB still exacts a devastating toll as about 50% of patients have metastatic disease with an overall survival (OS) rate around 30% [1, 2]. Although most of the patients are sporadic, a predisposition to this pediatric tumor may be inherited since a familial recurrence of NB has been observed in about 1% of all diagnosed cases [1, 2].

Recently, genetic determinants underlying the etiology of NB have been partially unveiled. NB shows a wide clinical and genetic heterogeneity: it can in fact be considered as an oligogenic disease that, differently from classic Mendelian diseases, results from poorly understood interactions among many genes [3]. While some of these genes may play a major role in the contribution to the disease, most of the susceptibility alleles that have been discovered up to now give only a small effect to the relative risk of developing NB. Hence, exceeding of a critical threshold for NB development is thought to derive from the sum of the limited effects of each gene involved [4], whereas the contribution of environmental participation to NB tumorigenesis is still unclear [5, 6].

In 2007 we first identified chromosome 2p as a genetic region candidate to harbor a NB predisposition gene by linkage analysis [7]. Thereafter, linkage analysis on a larger collection of families with recurrent NB, as well as other approaches, allowed discovering missense mutations of the Anaplastic Lymphoma Kinase (*ALK*), a major gene predisposing to NB [8–11] that maps on chromosome 2p. *ALK* is a receptor tyrosine kinase that proved to be a druggable target and is now exploited for novel therapeutic approaches [12–13]. Prior to the identification of *ALK* role in NB, mutations in another major gene, namely *PHOX2B*, which alone can be responsible for NB susceptibility, were discovered [14–15]. However, *PHOX2B* mutations are associated with syndromic NB that may present in association with other neurocristopathies such as Congenital central hypoventilation syndrome, and Hirschsprung's disease [16].

Additionally, genome-wide association studies led to discover multiple DNA polymorphisms within the genes *LINC00340* [17] *BARD1* [18], *LMO1* [19], *DUSP12*, *HSD17B12*, *DDX4*, *IL31RA* [20], *HACE1*, and *LIN28B* [21] that may play a critical role in NB and are involved in either low- or high-risk categories of the disease. Moreover, a recent outstanding study determined the genetic landscape of somatic mutations in high-risk NB by using a combination of whole-exome, genome and trascriptome sequencing [22]. Results from this study revealed a relative low percentage of recurrent somatic mutations in NB, being the most significant, in terms of frequency, those affecting *ALK* (9.2%), *PTPN11* (2.9%), *ATRX* (2.5%), *MYCN* (1.7%) and *NRAS* (0.83%) [22].

Despite the paucity of recurrent somatic mutations the complete spectrum of NB genomic alterations is yet to be fully discovered and it is now evident that each NB patient harbors a partially different set of either mutated genes or predisposing polymorphisms. In this light, we can argue that a personalized target-based medicine would therefore have to consider the peculiar genetic background of each NB patient in the future. Indeed, also rare variants affecting either coding genes or regulators of gene expression such as miRNAs should contribute to tumor susceptibility, aggressiveness and progression, although they may occur only in a small subgroup of patients or at least in a single case.

Among the genes emerged as associated to either predisposition or to the disease risk, *ALK* is a pivotal determinant of NB, since missense mutations of the tyrosine kinase domain of this membrane receptor recur in about 8–10% of sporadic cases and have been detected in almost half of the families with hereditary NB. However, *ALK* mutations segregating in these families show incomplete penetrance. For example, in the NB family considered in this study only 5/16 (31%) individuals, carrying the G1128A *ALK* mutation, actually developed NB. We thus hypothesized that mutations in other genes that might either modify ALK function or act as additional predisposing genes could be implicated in the emergence of the disease phenotype.

In this study we aimed to identify such gene(s) by performing whole-exome sequencing on two second degree cousins, both affected with NB, and on two unlinked healthy relatives belonging to a large family with NB recurrence.

## RESULTS

In order to decrease the number of polymorphisms that might be inherited by chance we sequenced the exome of two second degree cousins, who have a likelihood of 3% of sharing genomic variants, because of the genetic distance between them. Furthermore, we sequenced two unlinked relatives to discard those nucleotide variations in common with the two NB affected family members.

**Figure 1:**
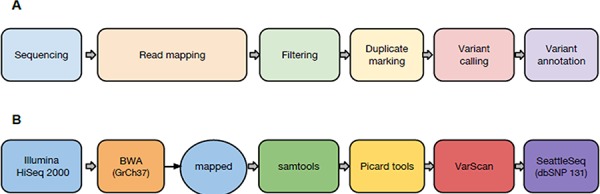
Pipeline schematic **(A)** conceptual workflow; **(B)** tools used in the present study.

After quality control, alignment, SNPs calling and annotation of the variants (Figure 1) our analysis reported an overall average of 70703 SNPs in the four DNA samples. Among all SNPs 51654 (73%) were intergenic or located in introns, and 19049 (27%) mapped within the coding sequence of genes (Table 1). We found 6999 nucleotide variations exclusively shared by the two NB patients and not detected in the two healthy relatives (Table 1). Of these candidate mutations 6744 were already described as common SNPs in the dbSNP database (http://www.ncbi.nlm.nih.gov/snp), whereas 255 were either novel or rare variations, showing a minor allele frequency lower than 0.01. Most of the latter variants were located outside of the coding sequence of genes or gave rise to synonymous changes as well. Thirty-two out of 255 variants were instead missense mutations, which involved 30 genes over the genome, and were considered for further analysis. As expected, among these variations we also observed the *ALK* mutation G1128A, already reported as segregating in this family [8]. None of the genes that were recently reported to be associated to either NB predisposition or to high/low risk cases [17–22] turned out to be mutated in this NB family.

**Table 1:** Statistics of whole-exome sequencing results

Variation Type	E5 (NB)	E10 (NB)	E6	E8	Shared by NB
**Missense**	7385	7032	8010	6624	662
**Synonymous**	8204	7678	8739	7317	739
**Nonsense**	77	75	84	72	9
**In splice sites**	32	26	31	21	3
**In a UTR**	2695	2763	2912	2345	284
**Intronic**	41457	44841	46277	36650	4404
**Intergenic**	9301	9163	10818	8111	808
**In dbSNP**	64952	67760	71348	57993	6744
**Not in dbSNP**	5172	4904	6700	3983	255
**Total SNPs**	70124	72664	78048	61976	6999

**Figure 2:**
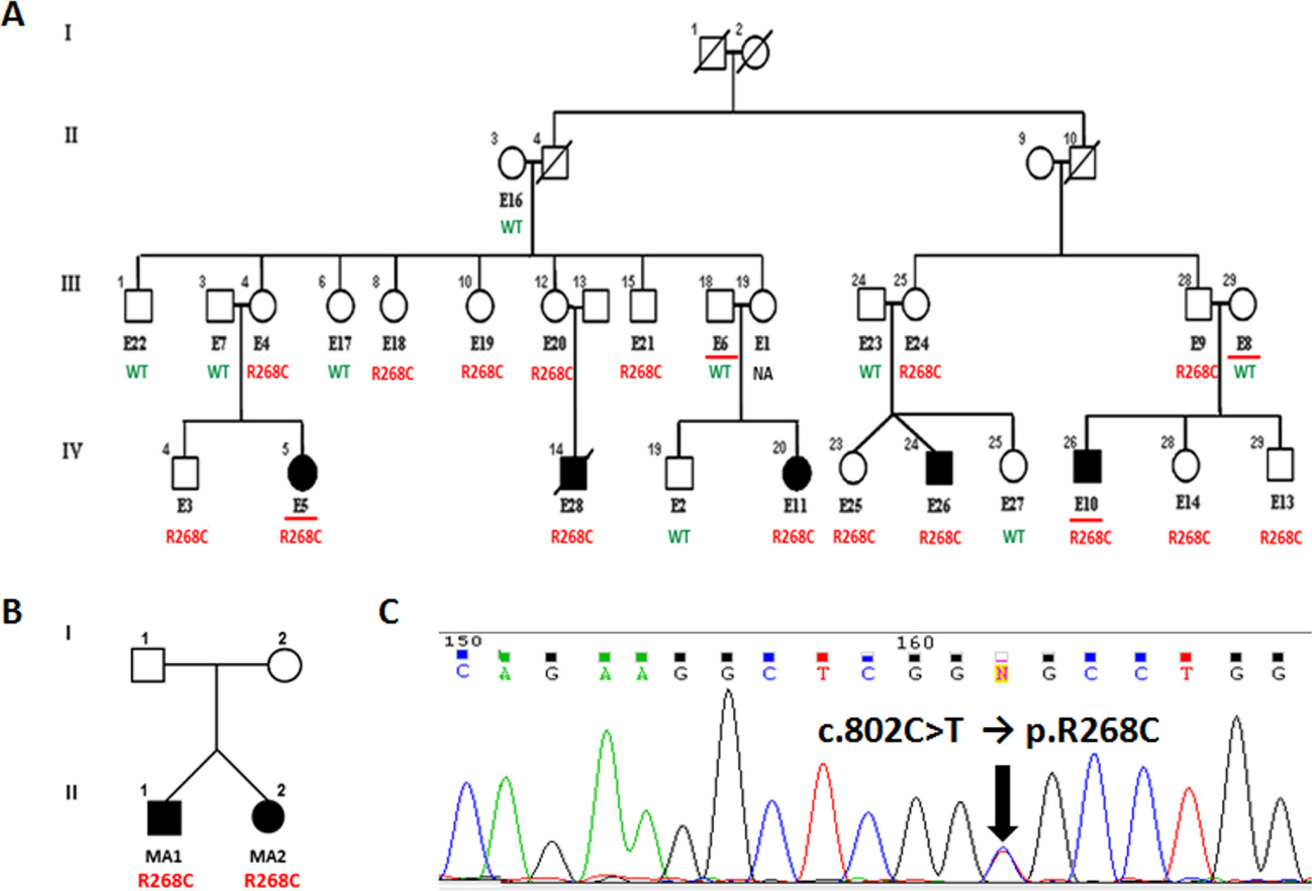
*GALNT14 c.802C > T* (R268C) mutation in NB **(A)** Pedigree of the NB family considered for mutation screening. All available individuals are coded with an E followed by a number. Family members analyzed by whole-exome sequencing are underlined. Germline segregation of the R268C *GALNT14* mutation is reported. **(B)** Pedigree of the small NB family with two heterozygous twins harboring a somatic R268C *GALNT14* mutation. **(C)** Example of the *c.802C > T* transition as observed by Sanger sequencing.

Variations in splice sites are those in the first two or last bases of the intron. Variations in introns exclude those mapping in splice sites. Family members are coded with an E followed by a number as in Figure 2.

Next, whole-exome sequencing results were validated by direct Sanger sequencing of the 32 missense variants identified. Conventional sequencing was performed on the same constitutional DNAs from the two NB affected family members previously considered for exome sequencing (E5 and E10, Figure 2A). As a result, 18 out of 32 germline variations were confirmed. Then, the analysis of the 18 validated mutations was first extended to all 5 individuals with NB belonging to the family, and secondly to the 20 other available family members, to identify those variants showing a proper segregation along the affected offspring. Actually, only 4/18 mutations proved to be shared by the 5 familial NB patients. These germline mutations are harbored by tumor protein p53 inducible protein 3 (*TP53I3*), polypeptide N-acetylgalactosaminyltransferase 14 (*GALNT14*), eukaryotic translation initiation factor 2-alpha kinase 2 (*EIF2AK2*) and BOC cell adhesion associated, oncogene regulated (*BOC*) genes (Table 2).

**Table 2:** PolyPhen-2 and SIFT scores for candidate variations

Chr	Position	RefBase	Sample Genotype	Gene	PolyPhen-2Score	SIFT Score
2	24300517	G	S(G/C)	TP53I3	Benign (0.004)	Tolerated (0.07)
2	31167749	G	R(G/A)	GALNT14	Probably Damaging (1.000)	Damaging (0.00)
2	37347242	C	M(A/C)	EIF2AK2	Probably Damaging (0.999)	Damaging (0.00)
3	112969587	G	R(G/A)	BOC	Benign (0.033)	Tolerated (0.05)

Prediction of the impact on protein function of the four variations segregating in the family with hereditary NB.

In order to predict the impact of these 4 variants on the protein function we employed both PolyPhen-2 (http://genetics.bwh.harvard.edu/pph2) [23] and SIFT (http://sift.jcvi.org) [24] softwares. Only mutations harbored by *GALNT14* and *EIF2AK2* genes were predicted as damaging, whereas both *TP53I3* and *BOC* variations were predicted as benign or tolerated SNPs (Table 2) and thus were not further investigated.

The two predicted damaging variations of *GALNT14* and *EIF2AK2* genes were then screened by Sanger sequencing in the constitutional DNA from the probands of 8 additional families with hereditary NB (Figure 2B, Supplementary Figure 1). Further Sanger sequencing was performed in tumor DNA of 167 randomly chosen sporadic NB cases, which showed at least a 70% tumor cell content and were stored in our laboratory. The *EIF2AK2* variant was not detected in any other NB patient. On the contrary, the *GALNT14* mutation was further observed in tumor DNAs of two heterozygous NB twins (Figure 2B) and in both somatic and germline DNA from one sporadic NB patient. This *GALNT14* mutation should segregate in the germline, however constitutional DNA from the twins and their parents was not available for testing. Finally, this *GALNT14* mutation was not detected in 132 unrelated healthy individuals.

According to human reference GRCh37, the *GALNT14* mutation we identified in NB cases maps on chromosome 2 at genomic position g31167749 of the forward strand (transcription of the gene occurs on the reverse strand) and is co-located with dbSNP rs143143842 (G/A). Referring to *GALNT14* isoform 1 (NM_024572.3 → NP_078848.2) the nomenclature of this mutation is: *c.802C > T* (transcript: ENST00000349752), that leads to a p.R268C (protein ENSP00000288988) amino acidic substitution in the encoded protein (Figure 2C). The 1000 genomes catalog of human genetic variations reports a Minor Allele Frequency < 0.01 for this heterozygous variation, which has been observed in only one individual from an African population (Yoruba in Ibadan, Nigeria) out of 2504 genomes analyzed. Moreover, the *GALNT14 c.802C > T* substitution has been reported as a confirmed somatic mutation in the COSMIC project (release 67) since it was found in a patient with colorectal carcinoma [25].

**Figure 3:**
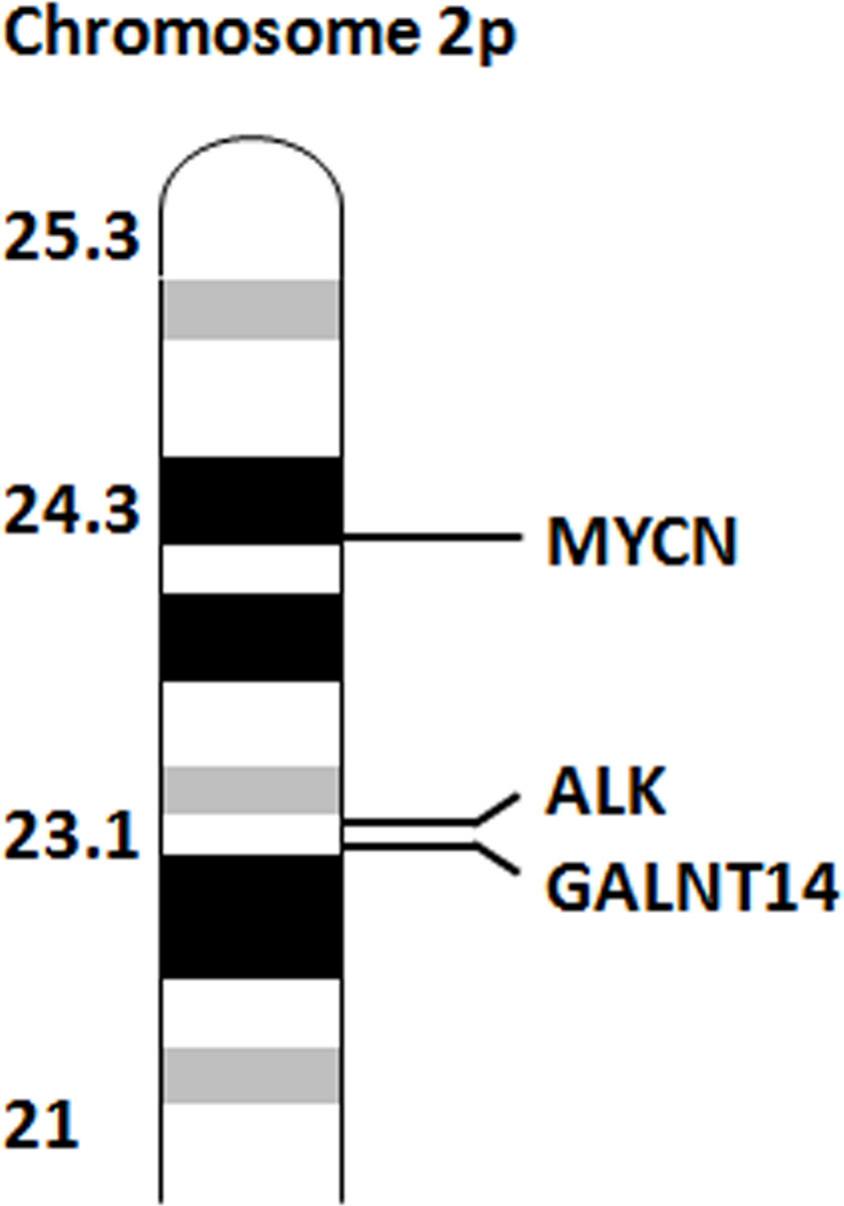
*GALNT14* maps close to *ALK* Representation of the relative distances of *MYCN*, *ALK* and *GALNT14* on chromosome 2p.

**Figure 4:**
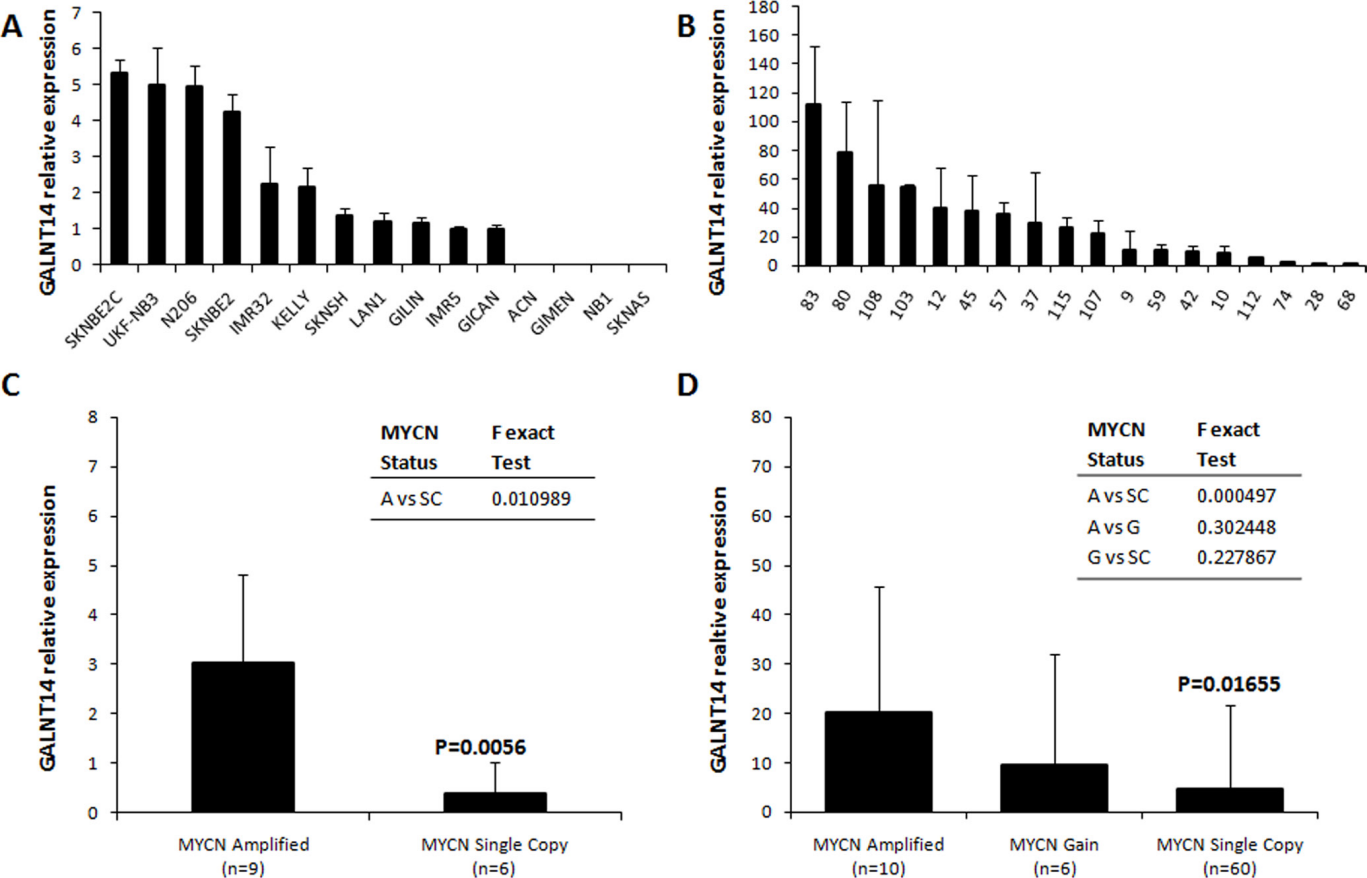
*GALNT14* mRNA expression in NB Expression of *GALNT14* in NB cell lines **(A)** and in *GALNT14* positive NB patients **(B)** by qPCR. **(C)** *GALNT14* average expression in *MYCN* amplified vs *MYCN* single copy NB cell lines. **(D)** *GALNT14* average expression in NB patients with *MYCN* amplification, gain and *MYCN* single copy. F is the value of the Fisher exact test between groups. *P*-values are relative to the statistical difference of *GALNT14* expression between *MYCN* amplified vs *MYCN* single copy groups and were calculated with the Mann-Whitney and T-Student tests in NB cell lines and patients, respectively. Y axis values are an arbitrary unit relative to the less expressed sample. A: *MYCN* Amplified; G: *MYCN* Gain; SC: *MYCN* Single Copy; *n* indicates the total number of samples for each group.

*GALNT14* maps closely to *ALK* on chromosome 2p23.1 (Figure 3), a genomic interval we had previously found out in linkage with NB in the family here considered for mutation screening [7]. Since *MYCN* amplification/gain may involve adjacent loci, including *ALK*, we hypothesized a possible extension of this genomic aberration to the *GALNT14* genomic region. We thus analyzed *GALNT14* mRNA levels by qPCR, that resulted expressed in 11/15 (73.3%) NB cell lines and 18/76 (23.7%) NB patients (Figure 4A, 4B). Noteworthy, a statistical significant association between *MYCN* amplification and *GALNT14* expression was observed in both NB cell lines and patients (Figure 4C, 4D).

**Figure 5:**
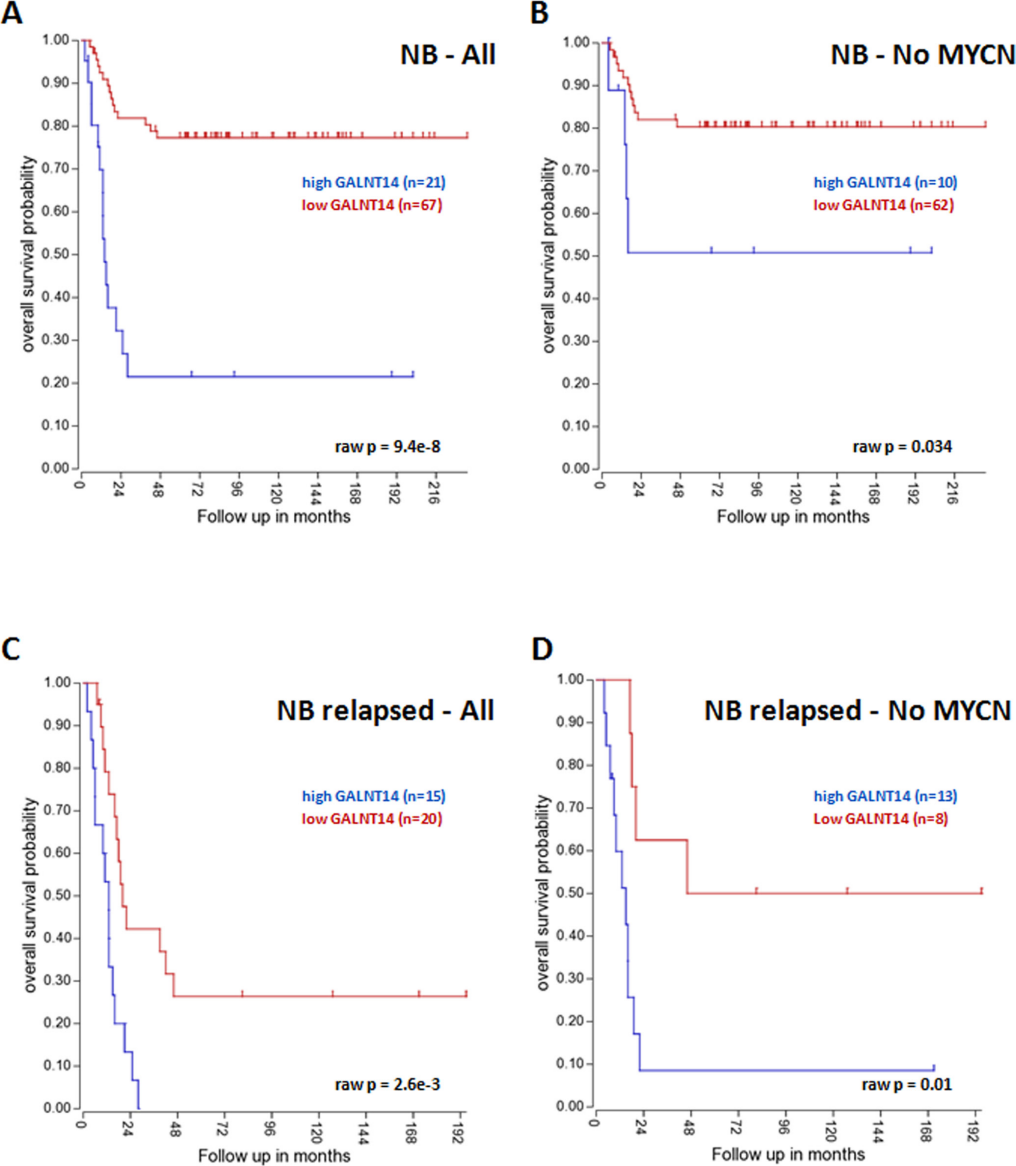
*GALNT14* impact on NB overall survival *GALNT14* higher expression is a negative prognostic factor for OS either when considering all NB patients **(A)** or only *MYCN* not amplified cases **(B)** *GALNT14* is a negative prognostic factor for OS in patients showing relapses or progression of the disease either when considering all NB patients **(C)** or *MYCN* not amplified cases **(D).** The cut off modus for *GALNT14* expression to draw Kaplan-Meier curves derives from the scan setting.

**Figure 6:**
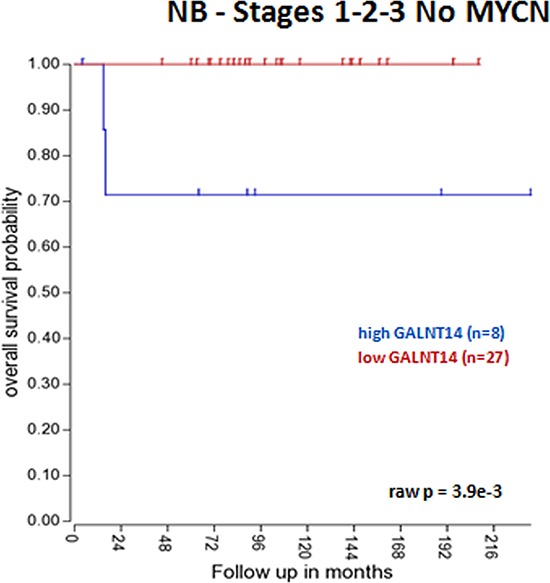
*GALNT14* impact on localized NB overall survival *GALNT14* higher expression is a negative prognostic factor for OS in NB stages 1-2-3 with no *MYCN* amplification. The cut off modus for *GALNT14* expression to draw Kaplan-Meier curves derives from the scan setting.

Interestingly, *GALNT14* higher expression significantly correlates with a worse OS in a public dataset of 88 NB samples (Tumor Neuroblastoma public - Versteeg - 88 - MAS5.0 - u133p2), either considering all NB patients (Figure 5A) or cases with no *MYCN* amplification (Figure 5B), which alone has a strong impact on the OS. Moreover, taking into consideration only NB cases who showed relapse or progression of the disease, a significant worse OS was observed when *GALNT14* expression is higher both in all cases and in *MYCN* not amplified patients (Figure 5C, 5D). Finally, we also observed an association of *GALNT14* higher expression with a worse OS in localized NB stages (stages 1, 2, 3) (Figure 6), whereas no association emerged for metastatic cases. OS data were analyzed using the R2 web application, which is publicly available at http://r2.amc.nl.

## DISCUSSION

Originally characterized by Wang and colleagues in 2003 [26], *GALNT14* is a gene that encodes a Golgi protein and is a member of the polypeptide N-acetylgalactosaminyl-transferase (ppGalNAc-Ts) protein family, which consists of at least 20 different isoenzymes [27]. These enzymes catalyze the first step in the biosynthesis forming the GalNAcα1-O-serine/threonine linkage in O-glycoproteins by transferring N-acetyl-D-galactosamine (GalNAc) to the hydroxyl groups on serines and threonines in target peptides of large proteins like mucins. The GalNAc-Ts are differentially expressed in cells and tissues and marked changes in expression are found in diseases including cancer [27]. Indeed, several human cancers show aberrant glycosylation, which influences multiple features of cell behavior such as apoptosis, proliferation and differentiation as well as migration and invasion.

For instance, GALNT3 was proposed as a marker of non-small cell lung cancer prognosis [28], whereas GALNT6 and GALNT14 itself were proposed as potential tissue biomarkers, since their high expression in breast and gastric carcinomas [29–32]. Two *GALNT5* somatic missense mutations were found in primary breast cancers [33] and functional testing revealed that these mutations reduced GALNT5 enzymatic activity [34]. In 2009 Guda and colleagues sequenced *GALNT12* in colon cancer cell lines detecting two somatic missense mutations in two different colorectal cell lines [34]. Both somatic mutations resulted to completely inactivate the GALNT12 enzymatic activity and fell within the GALNT12 catalytic and lectin binding domains, whose proper folding is required for GALNT activity [35]. Additionally, one nonsense and six more missense variants, which encoded essentially inactive GALNT12 enzymes, were exclusively found among colorectal cancer patients, suggesting that this gene has a role in this tumor. The authors concluded that their findings raised additional evidence that the aberrant glycosylation that is observed in colon and in other cancers may represent a primary abnormality resulting from mutations of GalNAc-T genes [34].

*GALNT14* over-expression was reported to play a critical role in cell migration, invasion and proliferation of breast cancer by promoting the epithelial-mesenchymal transition of breast cancer cells [36]. Because GALNT14 would contribute directly to malignant progression of breast carcinoma by altering the rate of cellular proliferation and promoting cell invasion and migration, this gene was suggested to be a novel therapeutic target for breast cancer [36]. Also, SNPs on *GALNT14* have been shown to be associated to the chemotherapy responses of patients with advanced hepatocellular carcinoma [37]. In other tumors such as pancreatic carcinoma, non-small cell lung cancer and melanoma *GALNT14* expression was observed to correlate with Apo2L/TRAIL sensitivity to proapoptotic signaling through O-glycosylation of Apo2L/TRAIL death receptors [38]. The authors suggested that O-glycosylation of DR4 and DR5 death receptors in various neoplasias modulates sensitivity to APO2L/TRAIL by promoting ligand-induced receptor clustering and consequent caspase-8 activation [38]. Apo2L/TRAIL is a member of the tumor necrosis factor superfamily, which recently has received considerable attention because of the finding that many cancer cell types are sensitive to Apo2L/TRAIL-induced apoptosis, while most normal cells appear to be resistant [39]. Interestingly, Apo2L/TRAIL signaling may represent a promising alternative in cancer therapies. Indeed, compounds such as the recombinant ligand and agonistic monoclonal antibodies directed against DR4 and/or DR5 are being investigated within clinical trials [40], and low concentrations of bortezomib sensitize different cancer cells, including hepatoma, colon and pancreatic cancer cell lines for TRAIL-induced apoptosis [41].

A possible involvement of *GALNT14* in NB pathogenesis is now suggested by our finding of an R268C mutation segregating in 2 out of 9 (22%) families with hereditary NB. Only one of the two NB families with altered *GALNT14* showed a concomitant recurrence of an *ALK* mutation. We can thus argue that GALNT14 should be an independent NB genetic determinant rather than a modifier of ALK. Aside *ALK*, which was already identified as the gene in linkage with NB in this family [8], our results strongly suggests that *GALNT14* should be linked to NB phenotype as well.

Although the R268C mutation seems to be rare we detected this variant also in one sporadic case with no *ALK* alterations, mainly supporting the link between *GALNT14* and NB. A larger screening of all *GALNT14* exons in a wide panel of NB sporadic specimens is needed to ascertain if other mutational sites affecting this gene are involved in NB, that would increase the rate of cases with *GALNT14* involvement. Also, the inactivating or activating nature of this mutation is yet to be determined and functional studies aimed at elucidating the molecular mechanism by which *GALNT14* acts in NB predisposition and/or progression are needed. The association of higher *GALNT14* expression with poor prognosis in a NB cohort would suggest that activating *GALNT14* mutations may contribute to NB pathogenesis and that further studies to evaluate GALNT14 as a potential candidate for therapy are warranted.

Recently, an association between another member of GalNAc-T protein family, namely *GALNT9*, and NB has been reported [42]. NB patients displaying expression of *GALNT9* in their tumor showed higher survival rates than patients with no expression. Particularly, *GALNT9* expression was proposed as a marker for positive outcome in the low-risk NB subgroup, whereas lack of its expression had a predictive value for tumor relapses [42]. Conversely, we found that a higher *GALNT14* expression in NB is associated with a worse OS that would suggest this gene as negative prognostic marker, especially in either localized cases or relapsed patients. This is also sustained by the association of a higher *GALNT14* expression with *MYCN* amplification, which may extend to *GALNT14* gene locus. Alternatively, we can hypothesize a possible functional regulation of *GALNT14* by *MYCN*. Finally, it is worth of note that also *GALNT13* has been found to be associated to NB as it was proposed as a marker of bone marrow involvement in stage 4 NB patients [43].

Conclusively, our analysis aimed to search for novel genes implicated in NB, revealing *GALNT14* as a novel candidate potentially involved in the disease predisposition. Although rare, the identification of a recurrence of a *GALNT14* mutation in NB adds evidence on an involvement of GalNAc-Ts in cancer generally, and in this pediatric neoplasia specifically. Indeed, an aberrant function of these enzymes can result in altered glycoproteins that in turn may be involved in the promotion of tumor aggressiveness. It is now evident that few, if any, recurrent mutations can be observed in a high percentage of cases, being those affecting *ALK* the most representative, involving about 8–10% of patients. Because of the low frequency of recurrent alterations the recruitment of a statistically significant number of eligible patients for future clinical trials will thus be a challenge. Notwithstanding that, personalized therapies will need to consider the whole genomic background of each patient, taking into consideration even the rarest mutations.

## MATERIALS AND METHODS

### NB families, patients and cell lines

Familial and sporadic samples were collected in our laboratory between 1996 and 2008. Germline genomic DNA was isolated from peripheral blood lymphocytes of both family members from 9 families with hereditary NB (Figure 2A, 2B and Supplementary Figure 1) and from the available sporadic NB cases, as well as from 132 unrelated healthy individuals. Somatic DNA was extracted from 167 sporadic NB specimens. DNA purification was performed using a standard phenol/chloroform protocol.

Name, origin and *MYCN* status of the 15 NB cell lines used in this study are listed in Supplementary Table 1. All cell lines were cultured in RPMI 1640 medium (Lonza) supplemented with 10% fetal bovine serum (Lonza), 1% Penicillin/Streptomycin (Lonza) and 1% L-glutamine (Lonza), under standard conditions.

### Whole exome sequencing

Germline DNA samples were sequenced in paired-end mode on the Illumina HiSeq 2000, obtaining over 120 million short (100 bp) reads. Starting from raw whole-exome reads, a modular open source bioinformatics pipeline was run to identify variations related to NB (Figure 1). After preliminary quality control of sequencing data by FastQC, sequences were aligned on the human reference genome (GRCh37) using the BWA aligner [44]. Reads flagged as secondary alignments and reads with mapping quality < 1 were filtered out with SAMtools [45]. Potential PCR duplicates were marked and removed by Picard tools. Variants were called by VarScan [46] on filtered alignments (with option --*p*-value 0.05) and annotated by SeattleSeq [47] using dbSNP v131. A set of high-quality variants characterizing the exome of NB individuals was obtained by discarding identified variants already present in public databases.

### Sanger sequencing

Sanger sequencing was performed for each missense variation identified by whole-exome sequencing. Specific primers for each variant were designed by Primer3 and blasted for specificity. PCR products were purified by ExoSAP-IT (GE-Healthcare). Bi-directional sequencing was performed by BigDye Terminator v1.1 kit (Life Technologies) on the ABI-Prism 3130 genetic analyzer (Life Technologies) and sequencing outputs were analyzed with the Sequencher 4.8 software.

### RNA purification and RT-qPCR

Total RNA was extracted from 15 NB cell lines and 76 NB samples using QIAzol Lysis Reagent (Qiagen) according to the manufacturer's instructions, and reverse transcription performed with iScript Reverse Transcription Supemix kit (Biorad). RNA quality was checked by 2100 BioAnalyzer using RNA 6000 Nano LabChip kit (Agilent Technologies) and quantified by NanoDrop (Thermo Scientific). Only RNAs with a RIN > 7 were included in subsequent experiments. The geNORM kit (Primerdesign) was employed to select the most stable housekeeping genes in our experimental set of samples that resulted to be *18S-RNA* and *GAPDH* in NB cell lines and *18S-RNA*, *GAPDH* and *UBC* in NB patients. RT-qPCR was carried out on the Mastercycler ep Realplex S (Eppendorf) using TaqMan FAM-labeled probes for *GALNT14* (Assay ID: Hs00226180_m1, Life Technologies) and for *18S-RNA*, *GAPDH* and *UBC* (Primerdesign).

## SUPPLEMENTARY FIGURE AND TABLE


