# Supplementary material for: Identification of GALNT14 as a novel neuroblastoma predisposition gene
Source: Oncotarget. 2015 Jul 3;6(28):26335–46. doi: 10.18632/oncotarget.4501 (PMC4694905; doi:10.18632/oncotarget.4501)
Supplement: Supplementary file 1 [file oncotarget-06-26335-s001.pdf]

SUPPLEMENTARY FIGURE AND TABLE

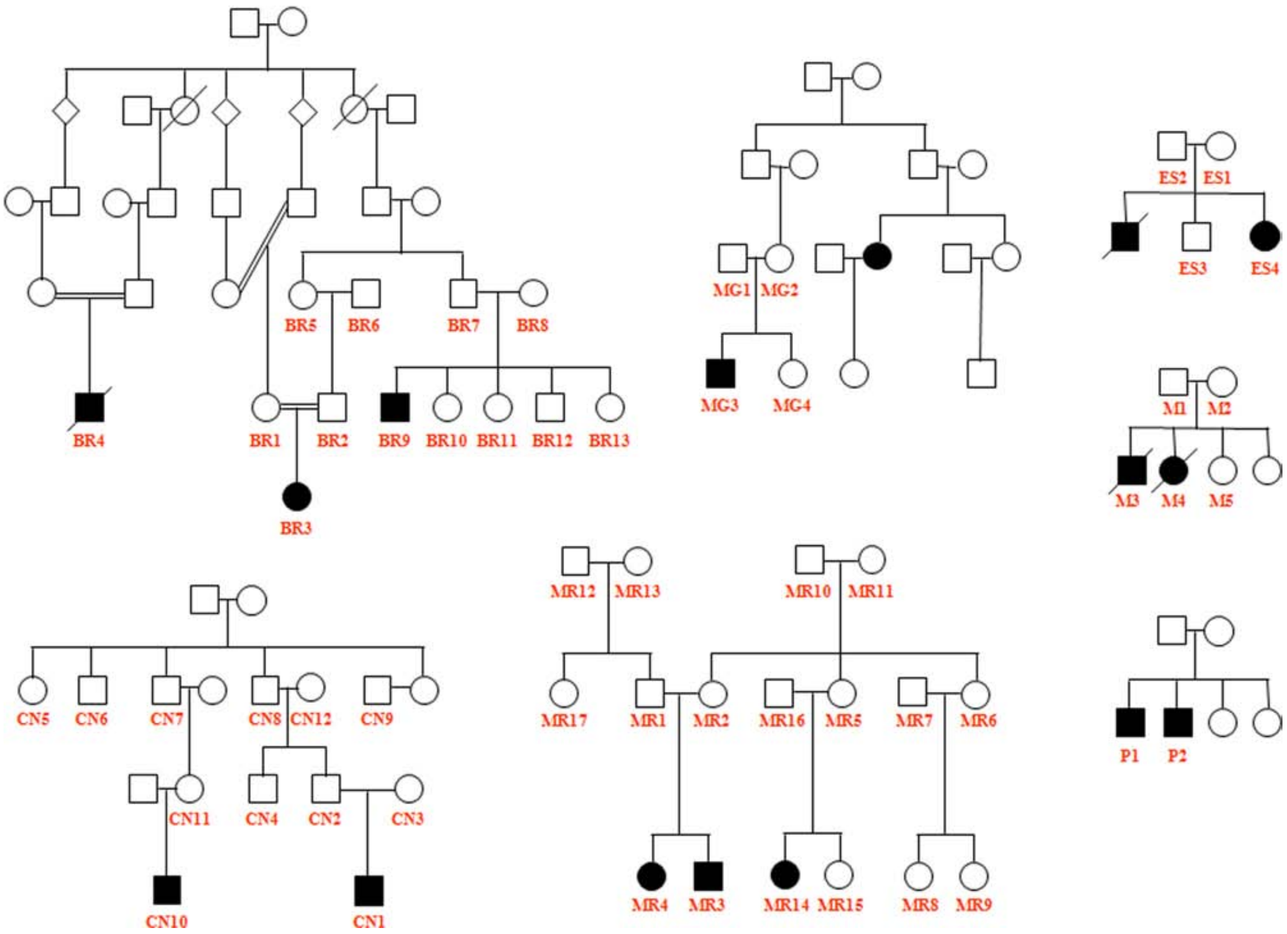

Supplementary Figure S1: Additional families employed in the *GALNT14* mutation screening. Overview of 7 additional pedigrees of families with hereditary NB that did not show the *c.802C > T GALNT14* mutation. Available individuals for analyses are indicated by a red code.

Supplementary Table S1: Cell lines information

Cell line	Origin	Tumor	MYCN Status
SK-N-BE2(C)	BBCF	NB	A
UKF-NB3	R. Luksch Lab	NB	A
N206	F. Speleman Lab	NB	A
SK-N-BE2	BBCF	NB	A
IMR32	BBCF	NB	A
KELLY	A. Quattrone Lab (ECACC)	NB	A
SK-N-SH	BBCF	NB	NA
LAN1	A. Quattrone Lab (ECACC)	NB	A
GILIN	BBCF	NB	A
IMR5	BBCF	NB	A
GICAN	BBCF	NB	NA
ACN	BBCF	NB	NA
GIMEN	BBCF	NB	NA
NB1	R. Luksch Lab	NB	NA
SKNAS	BBCF	NB	NA

BBCF: Biological Bank and Cell Factory, Core Facility of the IRCCS AOU San Martino-IST in Genoa (www.iclc.it); ECACC: European Collection of Cell Cultures. A: Amplified; NA: Not Amplified.
